# Implementing an Integrative Medicine Group Visit Facilitator Training Program to Improve Chronic Pain Management in the Safety-Net

**DOI:** 10.1177/27536130261453089

**Published:** 2026-05-20

**Authors:** Emiley Chang, Ruth Sie, Maria G. Mechure, Youngju Pak, Ryan Abbott, Tony Kuo, Christina Randle, Joann York, Ricky Chang, Allen W. Jang, Hope Cassano, Anish P. Mahajan, Ka-Kit Hui

**Affiliations:** 1 Department of Medicine, David Geffen School of Medicine at University of California, Los Angeles (UCLA), Los Angeles, CA, USA; 2 Department of Medicine, 21640 Harbor-UCLA Medical Center, Torrance, CA, USA; 3 117316 The Lundquist Institute, Torrance, CA, USA; 4 Center for East-West Medicine, UCLA, Los Angeles, CA, USA; 5 Department of Psychology, 21640 Harbor-UCLA Medical Center, Torrance, CA, USA; 6 UCLA Clinical and Translational Science Institute, Los Angeles, CA, USA; 7 School of Law, University of Surrey, Guildford, UK; 8 Los Angeles County Department of Public Health, Los Angeles, CA, USA; 9 Department of Family Medicine, David Geffen School of Medicine at UCLA, Los Angeles, CA, USA; 10 Department of Epidemiology, UCLA Fielding School of Public Health, Los Angeles, CA, USA; 11 14439 Department of Recreation Therapy, Rancho Los Amigos National Rehabilitation Center, Downey, CA, USA; 12 Department of Rehabilitation Services, Olive View-UCLA Medical Center, Sylmar, CA, USA; 13 Institute for Tai Chi and Qigong Arts, San Gabriel, CA, USA

**Keywords:** implementation and dissemination, integrative medicine, chronic pain, traditional Chinese medicine, tai chi, self-efficacy

## Abstract

**Background:**

As the opioid epidemic claims over 200 lives per day, there is an urgent need to increase access to evidence-based, non-pharmacologic modalities for pain management.

**Objective:**

To design, implement, and evaluate an integrative medicine group visit (IMGV) facilitator training program to support group visits incorporating mind-body therapies (eg, acupuncture, meditation, tai chi, yoga) for chronic musculoskeletal pain within the Los Angeles County Department of Health Services (LAC DHS).

**Methods:**

Prospective cohort pre-post study with mixed methods formative evaluation. Fifty-nine clinical staff from 3 LAC DHS medical centers and associated clinics enrolled in a 40-hour curriculum including online modules, case discussions, and onsite skills workshops in 2020. We used the *Self-Efficacy in Using Non-Drug Therapies for Common Symptoms* (SEND) scale to measure change in trainees’ self-efficacy to teach selected mind-body therapies for chronic pain management. We evaluated feasibility and acceptability with the course completion rate and obtained participant feedback through program evaluations and semi-structured interviews.

**Results:**

49% of staff members completed the course, primarily physicians (37%) and rehabilitation therapists (22%). Most participants (80%) were female and 28% more non-physicians completed the final exam than physicians (*P* = 0.040). SEND scores increased by 20.9 (*P* < 0.001). Trainees strongly valued the curriculum content, particularly the in-person tai chi and acupressure instruction, but recommended that the online modules be customizable to reduce time commitment. Disruptions from the COVID-19 pandemic affected course progress and completion rates.

**Conclusion:**

Overall, the IMGV facilitator training program was acceptable, feasible with program modifications under pandemic conditions, and increased staff confidence to teach others nonpharmacologic chronic pain management.

## Introduction

Chronic pain affects as much as a third of the world’s population, and causes major social and economic harms including decreased quality of life, lost productivity, and direct costs of medical care.^1^ Concerted efforts to aggressively treat chronic non-cancer pain in the 1990s dramatically increased the number, duration, and average dosages of prescriptions, quadrupling amounts of prescription opiates between 1999 and 2010.^2,3^ Unfortunately, increased opioid prescribing and marketing fueled an epidemic of opioid abuse, addiction, and overdose.^4^ A 2017 study found that nearly half of patients who died of opioid overdose had a diagnosis of chronic pain and had received opioid prescriptions within 30 days of death.^5^

In response, health care providers, policy makers, patients, and researchers are increasingly advocating for multimodal treatment strategies including non-pharmacologic modalities (NPMs) for chronic pain management.^6-8^ Integrative medicine includes NPMs and is well situated to address the multifactorial etiology of chronic pain—biologic, sociologic, psychologic, and environmental—as it is evidence-based, emphasizes patient-provider relationships, assesses the patient from a whole-person perspective, and “makes use of all appropriate therapeutic approaches, healthcare professionals, and disciplines to achieve optimal health and healing.”^9,10^

In 2017, The Joint Commission revised hospital performance measures for pain management to require accredited facilities to provide NPMs, such as acupuncture, osteopathic manipulation, physical therapy, massage therapy, and cognitive behavioral therapy.^11^ Evidence for the efficacy of mind-body therapies in chronic pain has been rapidly growing. In 2016, the National Center for Complementary and Integrative Health reviewed U.S. randomized controlled trials incorporating complementary approaches for pain management, such as acupuncture, manipulation, massage, relaxation techniques, tai chi, and yoga.^12^ The authors concluded that current evidence supports acupuncture and yoga for back pain, acupuncture and tai chi for knee osteoarthritis, massage therapy for neck pain, and relaxation techniques for severe headaches and migraines. The American College of Physicians subsequently included acupuncture, massage, and spinal manipulation as first-line NPMs for chronic low back pain,^13^ and the American College of Rheumatology conditionally recommended tai chi for knee osteoarthritis.^14^

Use of acupuncture has been steadily rising over the past 20 years,^15^ in part due to increased insurance coverage. In California, Medi-Cal (the state Medicaid program) provides coverage for acupuncture and chiropractor services for chronic pain. Starting in 2020, Medicare began covering acupuncture treatments specifically for chronic low back pain. However, the availability of approved providers remains a challenge, particularly in safety-net hospitals and clinics. Safety-net health systems share a mission to deliver essential care to low-income and vulnerable patients regardless of ability to pay or immigration status; they serve a significant proportion of uninsured and Medicaid patients and provide a disproportionate share of uncompensated care.^16^

Integrative medicine group visits (IMGV) that incorporate evidence-based NPMs are being increasingly utilized for chronic pain management.^17-21^ Some studies suggest IMGVs may result in lower pain intensity, reduced use of opioid medications, improved health related quality of life and sleep, as well as reduced depressive symptoms and loneliness.^22-25^ To address disparities in access to integrative NPMs, our study aim was to develop and implement a structured, scalable training for IMGV facilitators in a large safety-net health system.

## Methods

*Study Design.* We conducted a mixed-methods, prospective cohort pre-post study of an IMGV facilitator training program jointly developed by the Los Angeles County Department of Health Services (LAC DHS) and the University of California, Los Angeles Center for East-West Medicine (UCLA CEWM). The Institutional Review Board of The Lundquist Institute reviewed the study (reference #046783) and determined that it did not constitute human participants research.

*Setting and Participants.* We recruited a single cohort of staff trainees from 3 medical centers and the ambulatory care network within LAC DHS between January and September 2020. To be eligible, trainees had to provide patient education and be able to document patient encounters as part of their scope of work. We did not require prior experience with chronic pain management. We obtained recruitment support from the chief executive, medical, and nursing officers at each medical center as well as the director of the ambulatory care network, who requested nominations from departmental supervisors. As we planned to offer IMGVs in both English and Spanish, we encouraged recruitment of staff fluent in Spanish. We additionally sent a general email to hospital staff announcing the opportunity to participate in a free course to learn mind-body therapies and obtain training to lead future chronic pain management group visits.

*Intervention development*. The 40-hour certificate course was initially designed to include 20 hours of asynchronous online modules with didactic videos and pre-post quizzes (approved for continuing education credits for physicians, physical therapists, and social workers), seven 1-hour case discussions, 3 4-hour onsite skills workshops, and a 1-hour open book, multiple-choice final exam (Table 1). Supervisors approved onsite workshop attendance as part of scheduled training, while online modules and the final exam were completed outside of work hours. Several modifications to the course were later made in response to the COVID-19 pandemic (see *Intervention Delivery*).

**Table 1.** Planned Schedule for Integrative Medicine Group Visit Facilitator Curriculum

Week	Online modules	Case discussion	On-site workshop
[1] Opioid epidemic and intro to integrative medicine	Intro to opioid epidemic (10 min) Intro to integrative medicine (1 hr) Overview: Major traditional health systems (30 min) Overview: Major complementary modalities (30 min)	Integrative medicine physician	None
[2] Intro to traditional Chinese medicine (TCM)	Intro to traditional Chinese medicine (1 hr): *History, Modalities, Terminology, Theory, Diagnosis* Intro to acupuncture (1 hr): *History, Techniques and Mechanisms*	Acupuncturist	None
[3] Training videos: TCM & trigger points	Video: 10 key acupoints (20 min) Acupuncture for pain management (20 min) Video: Trigger point injections (20 min)	Acupuncturist	None
[4] Meditation in motion	Intro to tai chi & qi gong (1 hr) Video: Modified tai chi (20 min) Intro to mindfulness practices (30 min)	None	None
[5] Communication skills	East-West interprofessional communication (1 hr) Cultivating the therapeutic relationship (30 min): *Pain Empathy, Motivational Interviewing* Intro to group facilitator skills (30 min)	None	Tai chi #1 Acupressure #1 Group facilitation #1
[6] Stress, sleep, and mental health	Sleep & health (1 hr) Stress & resiliency (30 min) Integrative mental health (1 hr)	Psychologist: Group facilitation	None
[7] Nutrition & herbal medicine	Integrative nutrition (1 hr) Herbal medicine & supplements: Safety (1 hr)	None	Mindfulness stress reduction techniques Tai chi #2 Acupressure #2 Group facilitation #2
[8] Chronic pain management I	Chronic back pain & management (30 min) Self-care strategies for pain management (30 min) Opioid use disorder & chronic pain (1 hr)	Integrative medicine physician	None
[9] Chronic pain management II	Integrative approach to osteoarthritis (30 min) Exercise for chronic pain (30 min)	Integrative physical therapist	None
[10] Special populations	Integrative oncology (1 hr) Caring for the caregiver (30 min) Integrative medicine in the safety-net (30 min)	None	TENS demo Acupressure #3 Tai chi #3 Group facilitation #3
[11] Evidence-based medicine	Understanding integrative medicine research (1 hr)	None	Make-up or extra workshop (repeat #3)
[12] Final exam	Online final exam (1 hr)	Integrative medicine physician: Challenging case; exam answers	Optional CEWM clinic visit (3h): Acupuncture session, trigger points
[13] Evaluation	Program evaluation Curriculum feedback interviews (scheduled)	​	​

Acupressure and Tai Chi workshop sessions are led by an acupuncturist & tai chi practitioner, respectively.

A four-hour makeup session is offered for trainees who miss 1 session or require extra practice.

hr = hour; min = minutes.

Instructors affiliated with UCLA CEWM and LAC DHS were invited to create and deliver content based on their professional fields and areas of expertise; E.C. and K.H. reviewed and discussed all video module scripts to ensure that the level of detail was appropriate for a general professional audience and to reduce overlapping content. Online module topics prioritized topics covered in the UCLA CEWM integrative medicine fellowship curriculum, which specializes in integration of traditional Chinese medicine (TCM) with usual biomedical care. Discussion of other evidence-based integrative medicine modalities outside of TCM (eg, yoga, mindfulness meditation) were also included in online modules, case discussions, and workshops. As we did not require experience in chronic pain management to enroll, we added modules reviewing the basis of chronic pain, opioid use disorder, common types of chronic pain (eg, back pain, osteoarthritis), and self-care strategies. To complement the workshop trainings on group visit facilitation skills, an online module provided an overview of group medical visits, eg, benefits, differences from individual visits, explanation of the facilitator’s role, and examples of developing a group visit plan. A related module discussed the importance of therapeutic alliance in chronic pain management, defined pain empathy and compassion fatigue, and reviewed the 4 foundational processes in motivational interviewing.

We collaborated with a health psychologist (M.M.) experienced with group visits for chronic pain to develop a facilitator manual for the workshops which incorporated the framework from the Contra Costa County Regional Medical Center’s 2-day chronic pain group training program (completed by E.C.). Using Engel’s biopsychosocial model as a conceptual framework,^26^ our IMGV facilitator training program for chronic musculoskeletal (MSK) pain management addressed the interrelated etiologies of chronic pain: physical, emotional, and social. Trainees were taught modified tai chi, acupressure, and self-massage to address physical factors; stress reduction techniques and mindfulness practices to address emotional factors; and how to create a supportive group environment to address social factors.

Live group visit facilitation skills training components were delivered by M.M., E.C., and 2 DHS health psychologists. M.M. or E.C. led the group visit facilitation portions of the workshop sessions to allow trainees an opportunity to experience being in a group visit setting, review group visit facilitation concepts from the online modules, and practice approaches to empower and support patients to generate behavior change (∼1.5 hours per session). Content covered in the first workshop included setting group norms, collaborative agenda setting, identifying qualities of a group leader, SMART goals (Specific, Measurable, Achievable, Relevant, and Time-bound), and the importance of a trauma-informed approach, cultural sensitivity, and health literacy. The second workshop expanded on patient-centered communication skills and guided trainees in a partnered roleplay to practice motivational interviewing skills and create their own personal behavior change SMART goals. The third workshop reviewed topics from the first 2 sessions and trainees reflected on their personal behavior change projects. We described different types of disruptive group member behaviors that may threaten the goal of creating a safe and supportive environment and outlined strategies to respectfully manage the behaviors. Trainees then practiced these strategies in roleplays of a group visit session with different challenging scenarios, either as the facilitator or as a participant with a disruptive behavior. A health psychologist led a subsequent 1-hour live webinar case discussion on managing challenging scenarios that may arise during group visits and answered questions.

*Intervention delivery.* Recorded lectures, handouts, and supplementary reading materials were hosted on a UCLA online learning platform, while in-person course components were conducted at 3 of the LAC DHS medical centers. The course was originally scheduled to be completed over 13 weeks, but due to the COVID-19 pandemic trainees were allowed an extension up to 1 year. In response to the pandemic, the onsite workshops and case discussions had to be rescheduled and modified shortly after the first workshop and case discussion were conducted. The group facilitation and acupressure trainings were transitioned online, and tai chi instruction was conducted outdoors with screening, sanitization, and social distancing protocols in place. A half-hour break was incorporated to the workshop agendas to allow time for trainees to arrive at the tai chi instruction locations. Case discussions were conducted via online webinars and recorded for those unable to attend live sessions. Trainees were also asked to submit videos for baseline and final acupressure and tai chi evaluation in lieu of in-person evaluations as an additional safety measure; the time to complete these video assessments at home replaced the time originally scheduled for the seventh workshop. An acupuncturist and tai chi instructor taught the acupressure and tai chi portions of the workshops, respectively. Trainees were instructed to modify tai chi forms based on patient needs, eg, seated, stationary, multi-directional, and full movement.

*Data collection.* We conducted online assessments at course registration and at the end of training using REDCap surveys. Baseline demographic information included profession, medical specialty, years in practice, gender, and race/ethnicity. We also administered the CAM Health Belief Questionnaire (CHBQ),^27^ Maslach Burnout Inventory (MBI) - Human Services Survey for Medical Personnel (licensed from Mind Garden, Inc), and the Self-Efficacy in Providing Non-Drug Therapies to Relieve Common Symptoms (SEND) scale.^28^ At the end of the course, trainees completed a 50-question final exam on chronic pain and integrative medicine topics. Trainees also completed a program evaluation which repeated the SEND scale.

*Measures.* Our primary outcome was change in trainees’ self-efficacy to teach selected mind-body therapies for chronic MSK pain management using the SEND scale. We aimed to recruit 60 trainees, calculating a sample size of 42 would provide at least 80% power to detect the effect size of 0.45 (0.45 standard deviation (SD)) or more for pre-post comparison using a two-sided paired t-test with the level of significance of 0.05.^28^ As secondary outcomes, we evaluated change in trainees’ knowledge of integrative medicine, group visit facilitation, and chronic pain management based on module pre-post quizzes. We also assessed change in competency to teach modified tai chi and acupressure. Prior to the first workshop, trainees were asked to review a UCLA CEWM patient education brochure with diagrams and instructions on how to locate 10 acupressure points (based on the most common points used at UCLA CEWM clinics) and a tutorial video on the Tufts tai chi forms for knee osteoarthritis.^29^ At the first workshop, they were asked to demonstrate the acupressure points and tai chi forms based on self-study. After completing the training course, trainees submitted videos demonstrating the 10 acupressure points and the tai chi forms. Evaluators used a 3-point scoring rubric; trainees needed an average of 2.5 points with no scores of 1 to pass (Appendix A & B). Of note, the tai chi rubric was designed to focus on demonstrating an understanding of fundamental skills to lead instruction in group settings rather than mastery of specific forms, and the instructor emphasized that there are multiple tai chi styles that can achieve health benefits when performed safely.

*Data analysis.* The primary study hypotheses assessed mean score changes for SEND, tai chi skills, acupressure skills, and module quizzes before and after training. Continuous outcomes were analyzed using either a paired t-test or the Signed rank test for paired data, depending on the distributional assumptions. Additionally, a two-sample t-test or the Mann-Whitney test was used to compare the continuous outcomes between the 2 independent groups. Pearson’s correlation coefficient or Spearman’s rank correlation coefficient was used to explore relationships among continuous variables.

For multivariable analysis, linear regression models were used to predict post-training outcome measures using baseline score and potential covariates. Covariates considered included prior tai chi experience, prior acupuncture experience, gender, race/ethnicity (Asian heritage vs other), profession (physician vs other), and years in practice. Covariates showing statistically significant correlation with changes in the outcome were included in the final regression models. Residual analysis was conducted to validate model assumptions, and no significant violations were detected. Subscale analyses were performed, with Bonferroni correction applied to adjust *P*-values for multiple comparisons within each outcome. Reliability of the SEND scale was assessed using Cronbach’s α. All statistical analyses were conducted using SAS 9.4 (Cary, NC, USA).

We evaluated program acceptability and feasibility by administering a program evaluation survey and calculating the course completion rate. To identify factors that may impact dissemination and sustainability across the health system, E.C. conducted 30-60 minute semi-structured interviews with all trainees who completed the course. We discussed their experience with the program and curriculum, recommendations to facilitate training dissemination, and advice regarding potential barriers to IMGV implementation. Our study team reviewed transcripts to identify factors impacting course completion to guide future curriculum modifications.

## Results

A total of 59 trainees completed the baseline enrollment survey; Table 2 summarizes demographic characteristics and baseline measures. 47 (80%) of trainees were female and primarily from Asian American (41%), Latinx (29%), and White (25%) backgrounds; 3 trainees did not specify race/ethnicity and 1 identified as Black/African American. Enrollees had been practicing for 16 years on average and represented a broad range of disciplines including medicine (physicians, nurse practitioners and physician assistants), rehabilitation services (physical therapy, occupational therapy, recreation therapy, speech language pathology), social work, nursing, psychology, pharmacy, and health education. The 2 largest groups were physicians (37%) and rehabilitation therapists (22%). Some trainees indicated prior experience with tai chi (25%) and acupuncture (41%) or have recommended these modalities for pain management (46% and 61% respectively). Most had experience with yoga (71%), meditation (78%), and massage (69%) and have recommended these modalities for pain management.

**Table 2.** Participant Demographics and Baseline Measures

Baseline characteristics and measures (n = 59)	
Profession	Number (%)
* Physician*	22 (37.3)
*Physical Therapist*	9 (15.3)
*Social Worker*	6 (10.2)
*Recreation Therapist*	5 (8.5)
*Occupational Therapist*	4 (6.8)
*Physician Assistant; Nurse Practitioner*	2 (3.4)
*Psychologist*	1 (1.7)
*Nurse*	1 (1.6)
*Other*^a^	9 (15.3)
​	Mean (range)
Years in practice	16.4 (2, 41)
Gender	Number (%)
*Female*	47 (80)
*Male*	12 (20)
Race/Ethnicity^b^	Number (%)
*Asian American/Pacific Islander*	23 (41)
*Latinx*	16 (28.6)
*White*	14 (25)
*Multiracial*	2 (3.6)
*Black/African American*	1 (1.8)
*Did not specify*	3 (5.1)
Tai chi experience	Number (%)
*Yes*	15 (25.4)
*No*	44 (74.6)
Acupuncture experience	
*Yes*	24 (40.7)
*No*	35 (59.3)
Both tai chi and acupuncture experience	
*Yes*	8 (13.6)
*No*	51 (86.4)
​	Mean sum (SD)
CHBQ^c^	55.7 (7.6)
Maslach burnout inventory	Mean sum (SD)
*Emotional Exhaustion (EE),* n = 58	16.4 (8.7)
*Depersonalization (DP),* n = 57	4.2 (4.3)
*Personal Achievement (PA),* n = 57	38.4 (7.0)
Total sum^d^, n = 54	31.1 (16.3)

^a^Other professions: Physical therapy assistant, speech language pathologist, clinical pharmacist, health educator, health education assistant.

^b^Participants self-identified race/ethnicity using free text; responses were categorized by authors as above. White includes “Caucasian”; Asian American/Pacific Islander includes “Asian/Pacific Islander, Asian Pacific Islander, Filipino, Asian American, East Asian, Indian, Chinese, Asian Indian”; Latinx includes “Hispanic, Mexican-American”; Multiracial includes “Latino/White, Caucasian/American Indian.”

^c^CHBQ: CAM Health Belief Questionnaire.

^d^Total sum uses reverse scored PA subscale.

Most enrolled trainees demonstrated engagement with the curriculum; 50 (85%) participants interacted with the online video modules with a total of 586 hours delivered; 42 (71%) participated in the webinar case discussion series with a total of 184 sign-ins/views, and 38 (64%) attended at least 1 onsite workshop. 42 participants (71%) completed the baseline tai chi assessment and 41 (70%) completed the baseline acupressure assessment either at an onsite workshop or via video submission. Twenty-nine (49%) completed the final exam (released November 2020) and 28 (47%) completed the program evaluation between December 2020 and March 2021; 1 trainee passed the final exam but did not complete a program evaluation. The average training time completed per enrolled participant was 21.55 hours, representing 53.9% of the total 40-hour curriculum.

### Primary Outcome

Based on changes in SEND scores after the training, trainees had statistically significant improvements in self-efficacy for teaching patients and colleagues non-pharmacologic management of pain and other symptoms (*P* < 0.001) (Table 3). Change in SEND score was not associated with changes in acupressure, tai chi, or quiz scores, or the final exam score (Table 4). There were also no statistically significant correlations found between the change in SEND scores and MBI scores, including the 3 subscales. We confirmed the SEND scale demonstrated good internal consistency, with a Cronbach’s α of 0.96 (0.95 in the original study).

**Table 3.** Outcome Measures

Measure	Before training (SD)	After training (SD)	Mean difference (SD)	95% CI (*P*-value)
**SEND** (sum)				
Total sum	n = 59 57.1 (23.3)	n = 25 81 (11.9)	n = 25 20.9 (24.6)	10.8, 31.1 (*P* < 0.001)
*Teaching patients*	n = 59 30.4 (11.5)	n = 26 40.4 (6.1)	n = 26 9.2 (11.3)	4.6, 13.8 (*P* < 0.001)
*Teaching colleagues*	n = 59 26.6 (13.0)	n = 27 40.9 (6.4)	n = 27 12.7 (14.4)	7.0, 18.4 (*P* < 0.001)
**Tai chi skills**	n = 42	n = 29	n = 29	​
Total average	1.4 (0.2)	2.5 (0.3)	1.2 (0.4)	1.0, 1.3 (*P* < 0.001)
*Movements*	1.2 (0.4)	2.4 (0.6)	1.2 (0.6)	--
*Breathing*	1.6 (0.5)	2.9 (0.4)	1.2 (0.7)	--
*Balance*	1.5 (0.5)	2.4 (0.5)	0.8 (0.6)	--
*Mental Focus*	1.4 (0.5)	2.6 (0.5)	1.2 (0.6)	--
*Safety Awareness*	1.5 (0.6)	2.7 (0.5)	1.3 (0.7)	--
*Teaching: Demonstration*	1.4 (0.5)	2.3 (0.5)	0.9 (0.7)	--
*Teaching: Modifications*	1.2 (0.4)	2.6 (0.6)	1.4 (0.7)	--
*Teaching: Health benefits*	1.2 (0.4)	2.6 (0.6)	1.4 (0.7)	--
**Acupressure skills** (average of 10 acupoints)	n = 41	n = 29	n = 29	​
Total average	1.9 (0.6)	2.9 (0.1)	0.9 (0.5)	0.7, 1.1 (*P* < 0.001)
*Location*	2.0 (0.6)	2.9 (0.1)	0.8 (0.6)	--
*Landmarks*	2.0 (0.6)	2.9 (0.2)	0.8 (0.5)	--
*Indication*	1.7 (0.7)	2.9 (0.2)	1.1 (0.7)	--
**Module pre-post quizzes**				
% Correct: All participants (mean, SD)	n = 49 53.9 (14.7)	n = 48 72.2 (14.3)	n = 48 17.2 (17.6)	11.8, 22.3 (*P* < 0.001)
**Final exam scores**	N/A	n = 29 92.34 (7.75)	N/A	N/A
**Program evaluation**				
*Practice complementary non-drug therapies for chronic pain*	N/A	4.61(0.50)	N/A	N/A
*Teach complementary non-drug therapies for chronic pain*	N/A	4.54(0.58)	N/A	N/A
*Overall course quality*	N/A	n = 28 4.68 (0.48)	N/A	N/A

^a^Mean (SD).

**Table 4.** Associations With Change in SEND Scores

​	n	Pearson correlation coefficient (r)	Spearman correlation coefficient (r_s)_
Change in acupressure score	25	−0.05, *P* = 0.81	−0.13, *P* = 0.53
Change in tai chi score	25	−0.29, *P* = 0.16	−0.12, *P* = 0.57
Change in quiz score	25	−0.32, *P* = 0.11	−0.25, *P* = 0.23
Final exam score	25	0.19, *P* = 0.36	0.13, *P* = 0.53

### Secondary Outcomes

Table 3 summarizes statistically significant improvements in knowledge, competency to teach tai chi, and competency to teach acupressure. Overall, pre-post quiz scores for completed modules improved by 17% (*P* < 0.001). Total average tai chi scores improved from 1.4 to 2.5 (*P* < 0.001) as well as each skill component in the rubric (all *P* < 0.001 with Bonferroni correction for multiple tests). Total average acupressure scores improved from 1.9 to 2.9 (*P* < 0.001).

### Multivariable Analysis

For our multivariable analysis predicting post-training outcome measures, the final regression models included covariates showing significant correlation with the changes in outcomes (*P* < 0.1). Using a two-sample t-test, we found significant between-group differences for changes in SEND scores (profession: physicians vs non-physicians), acupressure scores (prior acupuncture experience: yes vs no), and tai chi scores (gender: male vs female, prior acupuncture experience: yes vs no, years in practice). We did not find any significant correlations among potential covariates for change in quiz scores. We obtained similar results with non-parametric tests using the Mann-Whitney test, with the exception that gender was no longer significant for tai chi scores.

We ran ANOVA with baseline scores and significant covariates (Appendix C). Based on the results from both parametric and non-parametric tests, we included profession as a covariate for final SEND score, prior acupuncture experience as a covariate for final acupressure score, gender and prior acupuncture experience as a covariate for final tai chi score. We did not include years in practice as a covariate in the model for tai chi score as it had minimal impact on the ANOVA results and R^2^. Overall, after adjusting for the baseline scores, none of the covariates were statistically significant except for the final tai chi score where females scored higher by 0.28 points on average (*P* = 0.03 after adjusting for the baseline tai chi score and prior use of acupuncture). Although we found a significant difference when comparing change in SEND scores between physicians and other health professionals, profession as a covariate was not significantly associated with final SEND scores after adjusting for the baseline scores.

### Factors Associated With Course Completion

We performed additional subgroup analyses for trainees who did not complete the course compared to those who passed the final exam with a passing score of 70% or higher as a marker of course completion. At baseline, 25% of trainees reported having personally experienced tai chi and 41% with acupuncture. Prior experience with tai chi was associated with final exam completion. Among trainees who had no prior experience with tai chi, 61% did not complete the final exam compared to 20% among those who did have prior experience; this difference was statistically significant (*P* = 0.007). When comparing professions, 28% more non-physicians completed the final exam than physicians, which was statistically significant (*P* = 0.040).

Trainees who completed the final exam had higher mean CHBQ scores than those who did not (*P* < 0.001). No statistically significant differences were found in overall MBI scores or its 3 subscales (using both sum and average); years in practice, prior experience with acupuncture, gender, and race (Asian American vs others) (Appendix D).

### Trainee Feedback

After completing the program, 28 trainees provided feedback via an online evaluation survey, which included open-ended questions on strengths, weaknesses, and areas for improvement. Additionally, 29 trainees participated in semi-structured feedback interviews discussing recommendations for training dissemination and implementation of chronic pain group visits within the LAC DHS health system. They were also invited to share examples of how they applied the knowledge and skills they learned during the course to their personal lives and patient care.

#### Acceptability and Feasibility

Trainees reported a high level of satisfaction with the quality of the curriculum, with an average rating of 4.7 on a 5-point Likert scale (Table 3). In the interviews, trainees consistently reported that the course exceeded expectations; aspects that they valued included level of organization, evidence-based content, and comprehensive scope. Instructors were noted to be highly qualified, supportive, responsive to questions and concerns, and able to accommodate a broad range of experience among the learners when teaching. Some trainees also expressed a sense of accomplishment and appreciated receiving a UCLA certificate and continuing education units.

Trainees also consistently highlighted the interactive tai chi and acupressure workshops as a major strength of the course; they emphasized the importance of receiving in-person instruction with real time feedback for effective learning. Similarly, they appreciated the group visit facilitation roleplay and practicing responses to disruptive behaviors. However, they had mixed opinions about the online format for the course modules. While they appreciated the flexibility of having a self-paced curriculum, they expressed a strong preference for in-person interactive learning and described initial technology issues with the online learning platform. We produced 1 podcast-style module to assess interest in this format compared to a traditional lecture-style presentation, but most trainees had no preference. Trainees also noted that completing the modules outside of work was a significant time commitment and the large amount of new information could be overwhelming, particularly on TCM topics.

While trainees expressed excitement about the opportunity to join the course, they also described the negative impact of COVID-19 related disruptions to their online curriculum progress and momentum. Because the online learning platform did not include a progress tracker, we provided individual progress report cards which were an effective form of motivation. Additional trainee recommendations included scheduling a midterm, providing notes or handouts for each module to facilitate exam review, and briefly summarizing relevant course content and key learning points at the start of each webinar case discussion to enhance trainee engagement.

#### Dissemination and Implementation

In the interviews, trainees shared insights on factors that may facilitate training dissemination and implementation. A common motivation for recruitment included learning new chronic pain management approaches to help patients who are not improving with standard treatment. Some trainees also wanted to activate patients to engage in self-management by teaching safe and low-cost methods for pain control. To motivate other staff members to enroll, they recommended obtaining buy-in through experiential sessions such as acupressure or tai chi and showing direct applications to patient care. To improve course completion rates, trainees emphasized the importance of having supportive supervisors who approved protected time to attend the skills workshops. They also recommended reducing the number of online modules to a core set tailored for different disciplines and shortening the length of each module. The remaining modules could be optional based on interest and/or scope of practice (eg, herbal medicine, oncology, nutrition, trigger point injections).

#### Applications

Trainees shared examples of how they applied new skills to their personal life and patient care -- particularly acupressure, tai chi, guided imagery, and breathing techniques -- for chronic pain and a range of other conditions. Those with little exposure to acupressure and tai chi prior to the course felt more confident and comfortable recommending them to patients afterwards. Some trainees practiced these skills with friends and family members; others incorporated new content into formal educational sessions and department in-services.

## Discussion

As part of the effort to increase access to evidence-based, nonpharmacologic approaches to pain management, the IMGV facilitator training program equipped an interdisciplinary group of health care workforce members to lead group visits within the second largest safety-net health system in the U.S. At the end of the 40-hour course, trainees were confident to teach patients and colleagues nonpharmacologic strategies to manage pain and associated symptoms and were qualified to teach 10 selected acupressure points and an evidence-based modified tai chi form for chronic knee pain.

Our pilot program contributes to the literature on dissemination of mind-body therapies for chronic MSK pain by describing a novel approach to implementation and evaluation of an IMGV facilitator training program in a large, resource-constrained safety-net health system with limited experience offering mind-body therapies. We strategically leveraged pilot funding and cultivated executive-level endorsement and support to systematically recruit trainees across multiple sites, including staff fluent in Spanish who can facilitate chronic pain IMGVs in languages other than English.^18^ We generated evidence that our IMGV facilitator training program is scalable and reproducible, flexibly accommodating real-world variability in clinical culture and resources as we sequentially implemented the training at each medical center. This program is also among the first to cross-train existing clinical staff from diverse disciplines—including rehabilitation therapists--to lead modified tai chi practice and acupressure during IMGVs, rather than relying on grant-funded instructors. Prior tai chi interventions for chronic pain had been led by professional tai chi instructors, which may not be a sustainable resource for safety-net institutions.^29-34^ The course also provided trainees with a broader foundation in TCM theory and terminology to improve interprofessional care coordination with acupuncturists outside the health system.

Acceptability was high based on trainees’ feedback on the curriculum and immediate practical applications and benefits to their professional and personal lives. To increase recruitment of co-facilitators, trainees recommended spotlighting the skills workshops and reorganizing the modules into required and optional lectures so trainees can tailor the curriculum to their interests and scope of practice. Evaluation of feasibility was complicated by the sudden changes to routine clinical services during the COVID-19 pandemic, which began shortly after staff started the course. Due to increased staffing demands during the COVID-19 pandemic, trainees who registered and completed the baseline survey may not have been able to start or finish all course components. Nevertheless, 49% of trainees successfully completed the training program following course modifications under pandemic conditions. Individual factors associated with course completion included higher baseline CHBQ scores, prior experience with tai chi, and non-physician profession. Higher baseline CHBQ scores suggests that trainees had a greater interest in integrative medicine and alignment with its principles. Similarly, trainees with prior tai chi experience may have been more likely to complete the course because taking a tai chi course as an active participant may indicate greater intrinsic interest in mind-body modalities, whereas prior experience with acupuncture as a passive treatment may not correlate with interest in learning acupuncture. It is unclear whether the association with profession was due to differential disruptions in staffing requirements during the COVID-19 pandemic vs underlying differences in interest in learning nonpharmacologic approaches to pain management due to education and scope of practice.

Factors that may limit generalizability of the IMGV facilitator training program’s results include the use of in-person instructors, trainee selection process, course interruptions due to the COVID-19 pandemic, and limited enrollment of nurses. Staff members were primarily recommended by supervisors, who tended to offer the opportunity to those who practiced mind-body modalities or expressed interested in chronic pain management. Challenges related to the COVID-19 pandemic changed the program structure and ability for trainees to complete the course as originally designed. The attrition rate was higher than anticipated, which reduced the effective sample size and may have reduced statistical power and influenced the analytic sample. While we were able to enroll a clinical nurse educator at 1 medical center, difficulty engaging nursing leadership at the 2 other medical centers was a barrier to recruitment of nurse champions.

Another limitation is that we did not evaluate trainees’ group visit facilitation skills during the brief roleplays. The skill and training of the facilitator is considered an individual-level determinant of group medical visit implementation,^35^ but there is a gap in the published literature on how trainee competency to facilitate group medical visits has been assessed.^36^ In general, trainee evaluations focused on change in confidence, knowledge, and attitudes and did not utilize competency-based tools to evaluate group visit facilitation skills.^37-40^ We had obtained additional funding to support the implementation and evaluation of pilot IMGVs and planned to have instructors observe and provide feedback to trainees after the visits. However, when group visits were halted due to the COVID-19 pandemic, we redirected this funding to produce a series of self-care management videos in English and Spanish. As a future development, we plan to reference IMGV facilitator guides from established programs as a standard for best practices^41^ and adapt validated tools from the mental health literature^42-44^ to evaluate trainees’ IMGV facilitation skills and link to patient-centered outcomes once IMGVs are implemented in our health system.

## Conclusion

Our IMGV facilitator training program to address chronic MSK pain has potential for broader implementation across LAC DHS and capacity building to provide integrative medicine services for chronic pain management. By investing in local champions to provide nonpharmacologic pain management services and train colleagues to lead integrative medicine group visits, this may improve comprehensive, multimodal pain management in the safety-net. IMGVs can help enhance effectiveness of other treatments such as medications, pain procedures and physical therapy by providing coaching and peer support. Given the experience of trainees applying learned skills to their personal lives, the program also has the potential to promote staff well-being.^45^ This collaboration between LAC DHS and CEWM serves as a promising model for community-academic partnerships, assisting vulnerable communities in leveraging whole system healing approaches to better health. Results and lessons learned about this program could be apply to other safety-net systems locally and across the United States. Plans for the future include IMGV implementation with evaluation of associations between group visit facilitation processes and patient-centered outcomes.

## Supplemental Material

Supplemental material - Implementing an Integrative Medicine Group Visit Facilitator Training Program to Improve Chronic Pain Management in the Safety-Net Supplemental material for Implementing an Integrative Medicine Group Visit Facilitator Training Program to Improve Chronic Pain Management in the Safety-Net by Emiley Chang, MD, MPH, MSHPM, Ruth Sie, DAOM, LAc, FABORM, Maria G. Mechure, PhD, LMFT, Youngju Pak, PhD, Ryan Abbott, MD, JD, MTOM, PhD, Tony Kuo, MD, MSHS, Christina Randle, CTRS, CBIS, Joann York, MPT, Ricky Chang, MD, MS, Allen W. Jang, ND, MA, Hope Cassano, DO, Anish P. Mahajan, MD, MS, MPH, Ka-Kit Hui, MD, FACP in Global Advances in Integrative Medicine and Health.

## Data Availability

The datasets generated during and/or analyzed during the current study are available from the corresponding author on reasonable request.*
