# Supplementary material for: Implementing an Integrative Medicine Group Visit Facilitator Training Program to Improve Chronic Pain Management in the Safety-Net
Source: Glob Adv Integr Med Health. 2026 May 20;15:27536130261453089. doi: 10.1177/27536130261453089 (PMC13191133; doi:10.1177/27536130261453089)
Supplement: Supplemental material - Implementing an Integrative Medicine Group Visit Facilitator Training Program to Improve Chronic Pain Management in the Safety-Net [file sj-pdf-1-gam-10.1177_27536130261453089.pdf]

Appendix A. Acupressure Training Evaluation Rubric

#	Acupoint	Location	Landmarks	Indication
1	GB 20			
2	GB 21			
3	CV/Ren 17			
4	P6			
5	LI4			
6	LI10			
7	SP10			
8	SP6			
9	ST36			
10	LV3			

Explanation of Scores

3: Consistently locates acupoints correctly and explains landmarks used. Demonstrates good understanding of >2 key indications for each acupoint.

2: Occasional errors in locating acupoints or may occasionally forget to use landmarks. Demonstrates understanding of 1-2 indications for each acupoint.

1: Frequent errors in locating acupoints or does not use landmarks. Does not know the indications for each acupoint.

Appendix B. Tai Chi Instructor Training Evaluation Rubric

The outcome-based criteria provided is meant to inform both instructor and participants with respect to learning expectations and specific assessment benchmarks. Provide feedback for scores less than 3.

COMPETENCY	3	2	1
Movements	Consistently performs TC forms with proper order, movements and upright body alignment; movements are slow, even, and continuous	Performs TC forms with occasional errors in order, movements and alignment; may occasionally vary speed and/or pause.	Performs TC forms with frequent errors in order, movements and alignment; frequent breaks in continuity.
Breathing	Consistently synchronizes breathing with TC forms, deep and slow	Breaths occasionally shallow, occasionally not synchronized with TC forms	Breaths are mostly shallow and not synchronized with TC forms.
Balance	Consistently transfers weight to	Occasionally forgets to transfer weight from	Moves from one TC form to the next

	control balance when moving forwards or sideways. Touches down first, then gradually transfers weight forward or backward.	one leg to the other, incompletely transfers weight, or abruptly transfers weight.	without transferring weight from one leg to the other.
Mental Focus	Consistently demonstrates calm, relaxed focus when performing TC forms	Occasionally appears uncertain or distracted when performing TC forms.	Appears uncertain, distracted, or hesitant when performing TC forms.
Safety Awareness	Consistently demonstrates awareness of position of body and arms with respect to other people in the room.	Occasionally lacks awareness of position of body and arms with respect to other people in the room.	Performs TC forms without consideration of position of other people in the room.
Teaching: Demonstration	Consistently explains each step of TC form clearly to learners before demonstrating and checks for understanding afterwards.	Occasionally errors in explanation of TC forms to learners; occasionally forgets to explain before demonstrating each form.	Frequent errors in explanation of how to perform TC forms or frequently forgets to explain before demonstrating.
Teaching: Modifications	Clearly discusses potential modifications for patients with different limitations (e.g. chair tai chi for disabling knee arthritis or paraplegia).	Mentions that patients may need individual modification of TC forms but does not explain how.	No mention of potential TC modifications for individual patients.
Teaching: Health	Displays full understanding of multiple evidence-based TC health benefits (e.g. mood, pain mgmt, stress mgmt, falls reduction)	Mentions 1-2 evidence-based TC health benefits.	No mention of TC health benefits.

Holistic Performance Rubric: Passing score is an average score of 2.5 with no "1" scores

3: Consistently performs breathing and TC form with proper movement, alignment, balance, and safety considerations while displaying an understanding of stress management benefits and etiquette.

2: Performs breathing and TC form with occasional errors in movement, alignment, balance, and/or safety considerations. Displays effort toward understanding the benefits of mind-body exercise and etiquette.

1: Performs breathing and TC form with errors and uncertainty. Has some difficulty performing proper movement, alignment, balance, and/or safety issues and seems unfamiliar with mind-body concepts.

Appendix C. ANOVA results

Final SEND Score

Parameter	Estimate	Standard Error	p-value
Intercept	75.33	6.34	<0.001
Baseline SEND score	0.19	0.10	0.08
Profession: Non-Physician	-7.67	6.00	0.21
Profession: Physician	--	--	--

R-Square	Coeff Var	Root MSE	Mean final SEND score
0.14	14.19	11.50	81.04

Final Acupressure Score

Parameter	Estimate	Standard Error	p-value
Intercept	2.73	0.10	<0.001
Baseline acupressure score	0.10	0.04	0.02
Prior acupuncture experience: No	0.02	0.05	0.61
Prior acupuncture experience: Yes	--	--	--

R-Square	Coeff Var	Root MSE	Mean final acupressure score
0.20	3.60	0.11	2.94

Final Tai Chi Score

Parameter	Estimate	Standard Error	p-value
Intercept	2.35	0.29	<0.001
Baseline tai chi score	0.03	0.18	0.88
Prior acupuncture experience: No	-0.15	0.09	0.09
Prior acupuncture experience: Yes	--	--	--

Gender: Female	0.28	0.12	0.03
Gender: Male	--	--	--

**R-Square**	**Coeff Var**	**Root MSE**	**Mean final tai chi score**
0.27	8.91	0.23	2.54

Appendix D. MBI and CHBQ by final exam completion status

**Maslach Burnout Inventory**		Mean (SD)
All enrollees	*Emotional Exhaustion (EE) (n=58)*	16.4 (8.7)
	Depersonalization (DP) (n=57)	4.2 (4.3)
	Personal Achievement (PA) (n=57)	38.4 (7.0)
	**Total sum**	**30.1 (16.3)**
Completed final exam (n=26-28)	*Emotional Exhaustion (EE)(n=28)*	16.9 (9.9)
	Depersonalization (DP) (n=28)	4.7 (5.2)
	Personal Achievement (PA) (n=28)	39.1 (6.2)
	**Total sum (n=26)**	**28.8 (18.5)**
Missing final exam (n=28-30)	*Emotional Exhaustion (EE)(n=30)*	16.4 (7.4)
	Depersonalization (DP)(N=29)	3.8 (3.1)
	Personal Achievement (PA)(n=29)	37.8 (7.6)
	**Total sum (n=28)**	**31.2 (14.1)**

*MBI total sum: Used reverse scoring for PA (higher is better) while EE/DP are negative (higher is more burnout)

**CAM Health Belief Questionnaire**	Mean (SD)
All enrollees (n=59)	55.7 (7.6)
Completed final exam (n=29)	58.9 (6.8)
Missing final exam (n=30)	52.5 (7.1)
